# Exploring techniques to distinguish between real images and those generated using stable diffusion XL

**DOI:** 10.1371/journal.pone.0339917

**Published:** 2026-01-27

**Authors:** Benjamin Sanders, David Morrison, David Harris-Birtill

**Affiliations:** School of Computer Science, The University of St. Andrews, North Haugh, St. Andrews, United Kingdom; University of Essex, UNITED KINGDOM OF GREAT BRITAIN AND NORTHERN IRELAND

## Abstract

The recent development of text-to-image diffusion models has allowed us to quickly generate realistic images from textual prompts. Despite enabling innovation in particular domains, concerns have been raised over the prospect of malicious users posing synthetic images as genuine. To assess if it is possible to discern between real images and those generated using diffusion models, a novel convolutional neural network was built, trained and tested on a bespoke dataset formed of authentic images from the ImageNet dataset and corresponding synthetic images generated using Stable Diffusion XL: an open-source text-to-image diffusion model. With the public release of this dataset, it is currently the largest publicly accessible collection of images generated using Stable Diffusion XL, significantly contributing to future research in this area. The positive results from our experiment performing a binary classification of synthetic and real images demonstrate the effectiveness in detecting synthetic images, with up to 98.38% accuracy using a ResNet-18 baseline, and 97.24% with the proposed CNN.

## Introduction

Generative artificial intelligence refers to a branch of artificial intelligence (AI) that focuses on producing content, frequently through the use of deep neural networks [1,2]. Generative Adversarial Networks (GANs) [3] were proposed by Goodfellow et al. in 2014 and are considered the first deep-learning model to generate realistic images that could not be detected by human observation [4]. Due to the ambiguity of the term “realistic”, metrics to rank the photorealism of the images produced by generative models soon followed - most notably the FID score [5]. High-performing GANs such as StyleGAN [6] propelled research into tools to detect when media had been produced by a GAN [7,8]. However, the limited domain of GANs and poor scalability [9] hindered the development of accessible interfaces that allow users to generate images easily.

Using diffusion models to generate images from textual prompts was first proposed in 2020 [10], and led to findings in 2021 stating that text-to-image diffusion models were “easier to scale and train than GANs” whilst also producing images of “more diversity” and a “better sample-quality” [9]. The broad domain and easy accessibility of text-to-image diffusion models make it possible that synthetic media—here used interchangeably with the terms “fake” and “AI-generated” to refer to images produced using artificial intelligence—will become more prominent in daily life, creating a demand for equally powerful tools to discern between real and synthetic media. Throughout this paper, the term “synthetic” is used primarily. Real images that have merely been doctored are not included in this definition. Bird and Lotfi [11] explored this by combining a set of real images from the CIFAR-10 dataset [12] and synthetic images generated using Stable Diffusion v1.4 [13], to form a dataset of 120,000 32 × 32 pixel images known as CIFAKE.

In this paper, a dataset with images at a higher resolution of 96 × 96 pixels was generated using Stable Diffusion XL [14], a version of the Stable Diffusion product developed by Stability AI. In this paper, “Stable Diffusion” refers to the family of text-to-image models, whereas “Stable Diffusion XL” denotes the specific version used to generate all synthetic images in this investigation. The dataset was coupled with a differing set of image classes taken from ImageNet [15], the larger, higher-resolution dataset from which CIFAR-10 originates. After curating real images and generating synthetic ones, a full dataset containing 100,000 images was compiled. After training and tuning a model using the dataset, the model produced a higher accuracy than the results reported by CIFAKE. With these positive results, the dataset has been released to the public to drive further research in this field. To the best of our knowledge, its 100,000 images make it the largest SDXL dataset by a significant margin. This dataset generated for the work in this paper can be found at: https://zenodo.org/records/10513773.

To further build on Bird and Lotfi’s results [11], the model was tested on image classes that were not used to train the model. These tests evaluated the model’s generalisability and found that it maintained a high accuracy, outperforming the more complex ResNet-18 model [16] when tested on the same dataset. The code, models, and instructions for running can be found at: https://github.com/benjamins5335/masters-project.

## Related work

**Fig 1:**
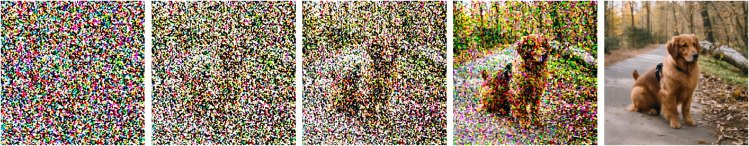
The process of generating an image using a diffusion model after (left to right) 1, 10, 20, 30 and 40 steps. Prompt was ‘a dog’. Interim images were extracted using modified code found in the GitHub repository in Introduction.

Diffusion models are a popular, effective method for generating images using AI. They performed better than industry-leading GANs despite their novelty [9]. These early yet promising results led to significant independent research into diffusion models for image generation, spearheaded by Ho et al. with their innovative paper “Denoising Diffusion Probabilistic Models” [10]. They studied a diffusion process used in thermodynamics first proposed by Sohl-Dickstein et al. in which a parameterised Markov chain progressively introduces noise to an image until it becomes entirely distorted [17]. They questioned whether they could reverse this process by training a neural network to take an image filled with Gaussian noise and use a U-Net [18] to remove it, leaving an image visually resemblant to a given text prompt. They found that the neural network struggled to produce an image resembling the textual prompt if they attempted to remove all the noise in one iteration. To solve this, they proposed reducing the amount of noise removed but increased the number of iterations and found their results significantly improved. The improvement after each iteration can be observed in Fig 1.

Alongside CIFAKE, there have been numerous other attempts to detect images created by diffusion models [19–23]. Wang et al. proposed DIRE, a paper that detects images generated by various diffusion models, but not SDXL. Training a convolutional neural network on real and synthetic images proved to be a reliable strategy, but many models still struggled to generalise to other datasets. Zhou et al. outlined methods to make images generated by diffusion models harder to detect [24] and Ho et al. proposed cascaded diffusion models that add more fine details after a typical diffusion process has finished, proving to lower the FID score even more [25]. Some studies chose to publicise their dataset to act as a catalyst for further research in the field. Zhu et al. outlined some of the largest datasets of this kind before releasing their own dataset of over 1 million images. Very few datasets comprised of images from Stable Diffusion XL, leaving a gap in research for this [26]. Bammey publicised a dataset of only 1,000 SDXL-generated images [27], while Li et al. have published a dataset of 12,000 images with real images with backgrounds generated by SDXL [28], which may be insufficient for research in detecting purely synthetic images. There is also a public dataset of 3,000 SDXL images of faces [29].

## Materials and methods

### Ethics statement

This research went through the University ethics approval process and written approval was provided by the University of St Andrews University Teaching Research and Ethics Committee (UTREC) with code CS17277. This includes the approval to use the ImageNet [15] dataset for non-commercial research and education purposes, as outlined by Stanford University and Princeton University in the Terms of Access on the ImageNet website. This research did not involve human participants, animals, or clinical trials.

### Dataset

Based on the results achieved by CIFAKE [11] with 120,000 images, a dataset containing 100,000 images (50,000 real and 50,000 artificial images each made up 10 classes of 5,000) was deemed sufficient for this research, and can be found at the following URL: https://zenodo.org/records/10513773. Samples can be seen in Figs 6 and 7.

#### Real.

When curating real images from ImageNet [15], the main requirement was to contain at least 5,000 images for ten clear, unambiguous classes, ideally of a high quality. CIFAKE [11], the most similar project to this one, used 32× 32 images from CIFAR-10 [12], a popular machine learning dataset curated from ImageNet. The quality and quantity of images in ImageNet made it a suitable choice for the project. However, extra steps had to be taken to select suitable images for the ten classes. ImageNet contains over 14 million images from over 20,000 classes called “synsets”. A synset could be downloaded by referring to its unique ID. However, only 1,000 IDs were made publicly available online [30]. The two options were to brute-force random IDs and manually inspect the contents or work with the smaller labelled set of IDs. The smaller set of IDs contained sufficient image classes, so the former option of brute-forcing did not need to be pursued. The email address listed on the ImageNet site was contacted to enquire about how to find a complete list of synset labels, but no response was received.

Furthermore, most synsets contained approximately 1,200 images, which falls short of the target of 5,000 images per class. As a result, the image classes formed in the dataset had to be broad enough to be covered by at least five synsets. Ensuring all synsets within a class had a visible resemblance was also important. For example, many synsets represented musical instruments, but there are few visual commonalities between a steel drum and a guitar, which would be detrimental to the training process.

#### Synthetic.

Between the popular 3rd party models to use, Stable Diffusion [13] was the choice over proprietary alternatives such as Midjourney and DALL-E 2 [31] for two primary reasons: its open-source model that enabled the code to be installed for free locally, reducing the dependency on cloud APIs and allowing amendments to be made to the code if necessary; and secondly, the findings from Borji [32] that concluded that the images generated by Stable Diffusion are generally more similar to real images than Midjourney and DALL-E 2. Using the best generative model would add more insight into the broader picture at the end of our investigation. Stable Diffusion, being open-source, created an active community of users, allowing for more insight into its usage compared to paid alternatives.

Stable Diffusion V1.4 (released August 2022) [33] was initially the favoured choice of model to use to generate the dataset, having been used in CIFAKE [11]; however, to make our research more novel and relevant, a newer version was used: Stable Diffusion XL. Released in July 2023, the authors claimed to offer “drastically improved performance” [14] and “next-level photorealism capabilities” [34] compared to previous Stable Diffusion models. The generation process was aided using pipelines imported from the diffusers Python library. This enables an image to be generated from a textual input within a Python file. The prompts were formed after research was conducted in the Stable Diffusion Discord server, an online public community discussing techniques on how to produce photorealistic and diverse images using various Stable Diffusion models [13]. To aid reproducibility, the schema for the prompts is detailed in the GitHub repository in Introduction.

**Fig 2:**
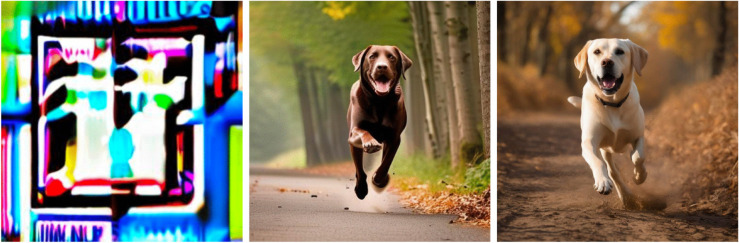
Comparison between images generated using Stable Diffusion XL. Images (from left to right) at 256× 256px, 512× 512px and 1024× 1024px. All images were generated using the prompt *“A photo of a labrador retriever dog, real-life setting running”*.

As Stable Diffusion XL [14] was optimised to create images at a resolution of 1024× 1024 pixels [35], the images were generated at this resolution despite the prior knowledge that the images would ultimately be downsampled to 96× 96 pixels. The pipeline did not allow images less than 256× 256 pixels to be generated. The authors’ claims were validated when the results generated at 256× 256 were, by inspection, much less photorealistic than images generated at 1024× 1024 pixels, as seen in Fig 2. These examples were generated after 40 inference steps with the configuration specified in the source code linked in Introduction.

Due to the higher resolution of images generated, generating 50,000 images was estimated to take 16 days on the allocated GPU, as the image generation process took a mean time of 27 seconds per image. To shorten this time, a portion of the image generation workload was delegated to an *NVIDIA DGX-1*, a machine highly optimised for large-scale AI tasks. This generated an image in a mean time of approximately 11 seconds. By splitting the load between the GPU and the *DGX-1*, a complete dataset was produced after seven days of parallel generation.

There was a trade-off when choosing the extent to which the Stable Diffusion XL [14] pipeline was fine-tuned. On one hand, a more photorealistic and visually diverse dataset would allow for a fairer comparison. On the other hand, it was important to test Stable Diffusion XL “as is” out of the box. When stripped back, this research is essentially human against machine. One could argue that excessive human input on the machine side may jeopardise the research. The prompts and variations diversified the dataset and made it as similar as possible to the real counterpart. Extra efforts could be made to make the datasets more visually similar, but with no guarantee that this would yield improved results and the desire to conduct an investigation as fair and reasonable as possible, the fine-tuning inputs were left to be more readable and reproducible.

### Neural network

Finding the best-performing architecture was an iterative process that involved many changes as the project progressed. Using a Convolutional Neural Networks (CNNs) are widely known for their high performance in image classification tasks [36]. The simple but high-performing architecture of CIFAKE [11] was chosen as a base in which we made some tweaks to the architecture, with the process for the final architecture being detailed in Grid Search.

#### Preprocessing.

**Fig 3:**
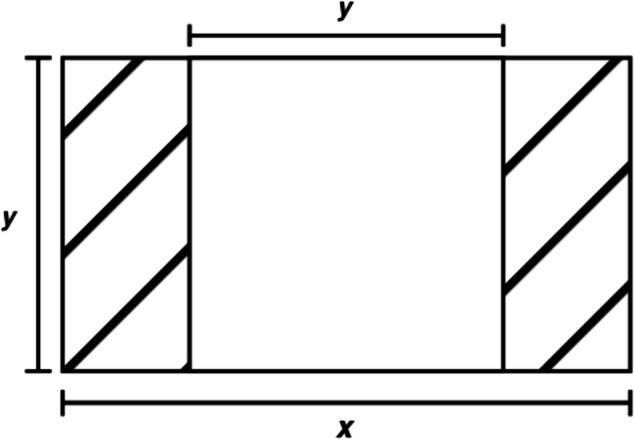
Illustration of cropping process. *y* is the height of the original image and *x* is the width. The longer dimension is reduced to the size of the shorter one (in this case, *y*) to form a square, with the equally-sized shaded areas on both sides being removed.

Once the images were generated, ensuring watermarking functionality was disabled, further manipulation was required to ensure the images were ready to be used to train the convolutional neural network (CNN). Watermarks were disabled by commenting out the contents of the apply_watermark function within the diffusers source code [37]. 1,000 real images were chosen from 50 ImageNet subclasses. Images exceeding 96× 96 pixels were cropped accordingly (Process detailed in Fig 3), while any images smaller than 96× 96 pixels were omitted.

A handful of the ImageNet [15] synsets contained less than 1,000 images. To ensure that each class had 5,000 images, the preprocessing script would use images from another subclass within that class. For example, the “thunder snake” and “ringneck snake” synsets contained only 809 and 921 images, respectively. To fix this, an extra 270 images were used from the “green snake” synset to bring the total number of snake images up to 5,000. When there was a surplus of images in a synset, the first 1,000 images of a resolution greater than or equal to 96× 96 pixels were used. Any remaining images were ignored.

Once a dataset containing 100,000 images was curated, the next step was to downsample the entire dataset using a consistent, deterministic downsampling method. The OpenCV library enabled this to be easily implemented. Fig 3 outlines the process of cropping the images.

All downsampling methods were implemented in the preprocess.py file, which takes a path to the folder containing the raw images as an input and outputs the downsampled dataset, separated into test and train folders. A train/test split of 80/20 was used as this has been deemed the industry standard for a dataset of a medium size [38].

#### Grid search.

A systematic approach was taken to build, train and fine-tune the CNN. The initial objective was to use existing architectures and minimal hyperparameter tuning to ensure the model’s functionality. Given the similarity of the problem being addressed, the architecture described in CIFAKE was considered an appropriate baseline for initial experimentation [11]. During architecture selection, it was determined that shallower networks provided higher accuracy when detecting synthetic images, as deeper ones tended to overfit to SDXL images.

**Fig 4:**
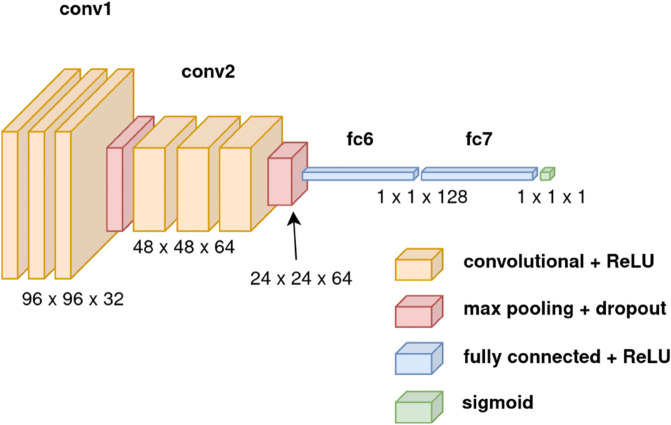
Architecture of the final classification model. Modified from [43].

The final experimental architecture is shown in Fig 4. This architecture had three convolutional layers, followed by a max-pooling layer. This was followed by three more convolutional layers and another max-pooling layer. After this, the input was flattened and passed through 2 fully connected layers before the result was set to fall between a range of 0 and 1 by using a *Sigmoid* layer. This architecture was shallow compared to popular models such as ResNet-18 [16], but tests with deeper models resulted in more overfitting, with a bigger gap between the validation and training accuracy. Hence, the better-performing shallow architecture was chosen for the final model. Furthermore, this eased the computational load on the GPU and allowed for faster training. Following this, a grid search was run to assess which combination of hyperparameters performed the best, as seen in Table 1 (Table 2).

**Table 1:** The hyperparameters included in the grid search and their values. The ranges were informed by values taken from relevant literature cited in the table.

Learning Rate	Batch Size	Epochs	Dropout
0.00001 [39]	16 [40]	10 [41]	0.0 [42]
0.0001	32	30	0.2
0.001	64 [40]	50 [41]	0.4 [42]
0.01 [40]			

**Table 2:** Layer-wise configuration of convolution and pooling operations.

Layer Type	Kernel Size	Stride	Padding
Convolutional (all)	3 × 3	1	1
Max Pooling (all)	2 × 2	2	0

**Fig 5:**
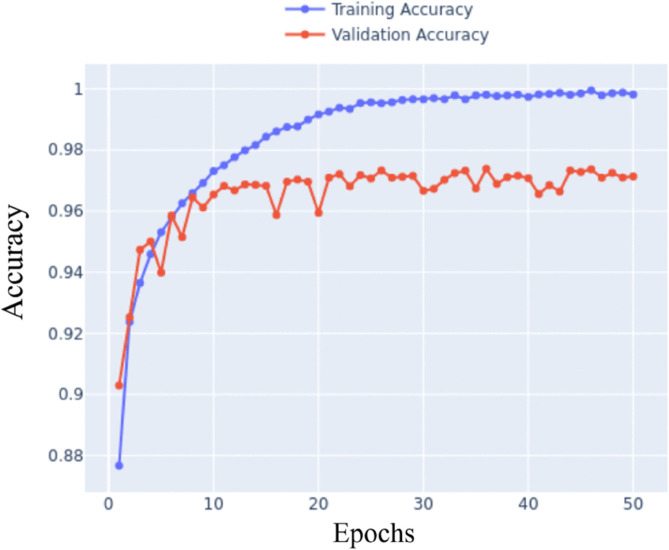
Training curve detailing convergence over time.

Once the grid search had concluded, attempts were made to find any patterns with the validation accuracy towards the end of the training process. The best results came from models trained with a dropout rate of 0.4, the highest value in the grid search. This left an open-ended question about whether a dropout value greater than 0.4 would produce better results. As a result, a second grid search was run with a more complex architecture with additional convolutional layers. A constant batch size was used as that had little effect on the validation accuracy. 50 epochs was also a constant as it was observed that all tests converged before the 50 epoch mark as stated in Fig 5. The full list of parameters that were used are shown in Table 3.

**Table 3:** The hyperparameters included in the grid search and their values. The range for dropout was informed by values taken from relevant literature cited in the table.

Learning Rate	Batch Size	Epochs	Dropout
0.0001	32	50	0.4 [42]
			0.45
			0.5
			0.55
			0.6
			0.65 [42]

The results from the second grid search enabled a rudimentary understanding of the hyperparameter combinations that yielded more positive results. From here, manual hyperparameter tuning was carried out, where combinations similar to the high-performing grid search configurations were tested. The hyperparameters for the final model are shown in Table 4. The full architecture can be found in the binary_classifier.py file in the GitHub repository in Introduction.

**Table 4:** The hyperparameters used to train the final model.

Learning Rate	Batch Size	Epochs	Dropout
0.0001	32	50	0.5

## Results

### Examples from dataset

See Figs 6 and 7.

**Fig 6:**
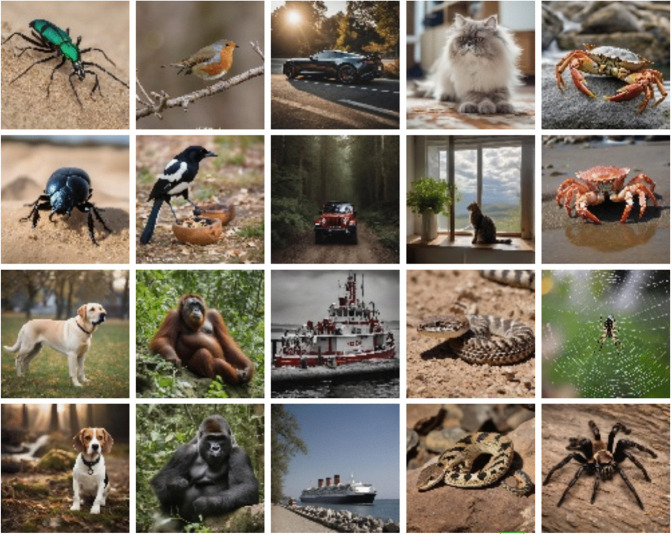
Subset of Images Generated with Stable Diffusion XL [14].

**Fig 7:**
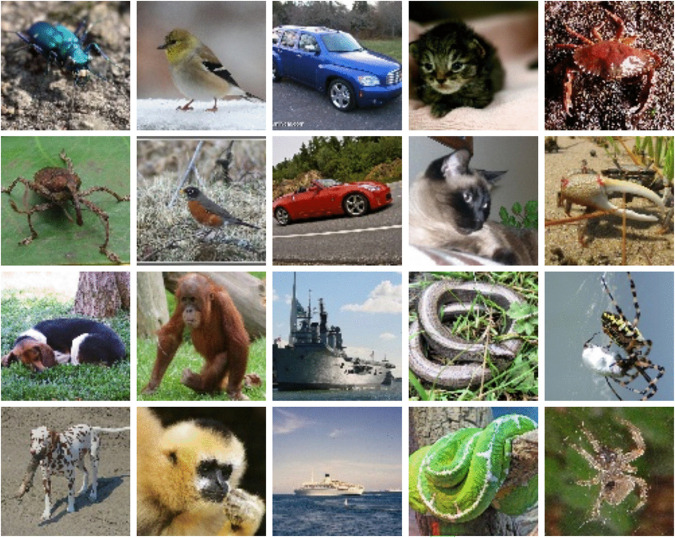
Subset of Real Images Taken From ImageNet [15].

### FID score

Two FID scores [5] were calculated and can be found in Table 5. Between the synthetic dataset and the real dataset, the score was 24.31, a score significantly better than any of the datasets produced by Borji [32] in his paper assessing the quality of datasets generated using diffusion models, with the lowest FID score reported by Borji being approximately 35%. To our knowledge, there is no public dataset with a lower published FID score, as of September 2025. As a result of this, Borji’s FID score acted as a benchmark, which was improved upon by our dataset, and outperforms other SDXL datasets in both visual realism and size. Despite the popularity and wide preference of the FID score as a metric, it cannot be seen as a definitive assessment of visual realism [44], due to the failure to capture distortion levels and varying results based on sample size.

**Table 5:** FID scores for dataset comparisons.

Comparison	FID Score
Real vs. Synthetic	24.31
Train vs. Test	1.40

Furthermore, the FID score of 1.40 between the train and test sets was very low, given the lowest score one can achieve is 0. This proves the similarity between the training and test sets, adding more integrity to the test results produced by the final model outlined in Test Set.

### Test set

#### All classes.

Table 6 shows the confusion matrix for both the original model on the test set. The original model produced an accuracy of 97.24%, a significant improvement on the 92.98% accuracy produced from CIFAKE [11] research. As seen in Table 7, the ResNet-18 [16] model outperformed the original model by achieving an accuracy of 98.38%. This is likely due to the deeper architecture of ResNet-18. Its 18 layers make it more capable of detecting features that models with smaller architectures may miss. The original model sacrifices this capability to allow for shorter training times. The accuracy from both models was less than that of the results produced in GenImage [26], where the ResNet-50 model trained on Stable Diffusion-generated images was able to detect them with 99.9% accuracy, but that is to be expected given the more increased depth of ResNet-50.

**Table 6:** Confusion matrix for test set.

		Actual	
		Real	Synthetic
**Predicted**	**Real**	9618	170
	**Synthetic**	382	9830

**Table 7:** Performance metrics for test set for Original Model and ResNet-18 pre-trained model.

	Original Model	ResNet-18 [16]
**Accuracy**	97.24%	98.38%
**Precision**	98.26%	98.41%
**Recall**	96.18%	98.35%
**F1 Score**	97.21%	98.38%

Precision and recall are more important metrics in scenarios where the cost of false positives and false negatives differ in a classification problem [45]. With our problem, we deemed there was no clear evidence to suggest that the cost of a real image being perceived as synthetic differs from that of a synthetic image being perceived as real; both outcomes have equal consequences in real-world scenarios. As a result, precision and recall can be treated as equally important metrics. The balanced distribution of classes in the test set means the utility of the F1 score was lessened but still provided insight into the presence of false positives (a synthetic image that the model predicted to be real) and false negatives (a real image that the model predicted to be synthetic). Accuracy was deemed the most important metric due to its equal consideration of true positives and true negatives.

### Subclasses

**Fig 8:**
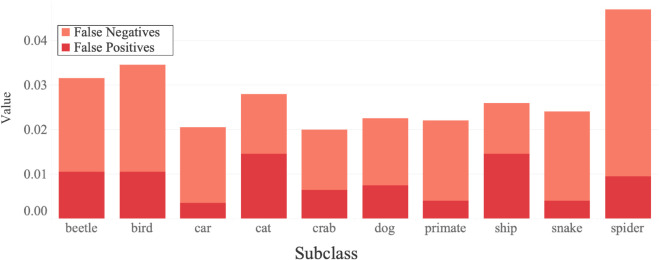
A breakdown of the incorrect predictions by subclass (5000 images per subclass).

After a deeper dive into the model’s performance, it was observed that the model found certain subclasses easier to classify than others, as seen in Fig 8. Notable observations included the proportionally larger number of false negatives for the spider class compared to the rest of the test set. Other classes that contained smaller, more intricate subjects such as beetles or birds also struggled more, suggesting a limitation with the model’s ability to detect generally smaller objects. The model performed well when classifying cars, with only seven false positives from the test set of 2000 images. As observed in Table 6, false negatives were more common than false positives with the original model. This is reflected across each subclass as seen in Fig 8.

## Generalisability

An important consideration is the model’s performance on unseen data. To assess the generalisability of the model, an additional dataset was created containing real and synthetic images of guitars, fish and rodents, and subsequent tests were run using the original model. The real images were taken from the “acoustic guitar” synset with ID n02676566 and the “electronic guitar” synset with ID n03272010 on ImageNet [15]. The synthetic images were generated using the same method as the rest of the artificial dataset, where a handful of additional keywords were added to diversify the dataset. These included “on wall” and “being played” amongst others. The final set comprised of 5656 real images, and 5656 synthetic ones. Tables 8 and 9 show the positive results on the unseen data, with an accuracy of 92.04% for the original model outperforming both the results of 92.98% from the CIFAKE paper [11] and the 88.89% accuracy produced by the ResNet-18 model [16]. The fine-tuned ResNet-18 model may have overfitted to the features of the training set, resulting in lower accuracy.

**Table 8:** Confusion matrix for original model’s performance on the unseen set.

		Actual	
		Real	Synthetic
**Predicted**	**Real**	5544	788
	**Synthetic**	112	4868

**Table 9:** Performance metrics for unseen data compared with ResNet-18 model.

	Original Model	ResNet-18 [16]
**Accuracy**	92.04%	88.89%
**Precision**	87.56%	82.19%
**Recall**	98.02%	99.30%
**F1 Score**	92.50%	89.94%

On the tests on the unseen data, the recall is much better than the precision for both models tested. This is likely a result of the real dataset having a greater visual diversity - the poses and angles of the subjects in the real dataset are more unique than those of the SDXL dataset. Adding variant keywords to the Stable Diffusion XL [14] prompts helped improve the dataset, but it would be challenging to manipulate the inputs enough to create unique scenarios for the 5,000 images in each class. The greater variance in the real dataset could be why classifiers perceive unseen data as real more often than synthetic. ResNet-18’s [16] poorer performance on the unseen dataset was not anticipated but could be attributed to its more complex architecture, making it more sensitive to the characteristics of the initial training set.

When determining the extent of the generalisability of the model, it is essential to consider various factors which may hinder the model’s performance. Many applications and services besides Stable Diffusion XL [14] can generate synthetic images. Furthermore, various parameters can be changed within each application to affect the image generation process, ultimately resulting in an image with unique attributes.

## Limitations and future research

While our research presents novel discoveries, there are limitations that inform directions for future work. Firstly, the model struggled with classes containing smaller objects such as spiders, birds and beetles. Further research could also be carried out into the models general performance across different classes outside of images of guitars, rodents and fish. Explanability can also enhance our claims stated in this paper, but due to the inherent opaqueness and black-box nature of the model as well as the prioritisation of other areas to enhance our research, this was not explored in further detail. There is also opportunity for further research to experiment using newer, better-performing models than ResNet-18, as this was chosen due to its strong performance at this time the research was carried out.

Secondly, this research is only relevant to images generated using Stable Diffusion XL, which is one of many text-to-image diffusion models publicly available. Research has been carried out on other open-source Stable Diffusion models, but less so on proprietary models such as Midjourney and Dall-E due to the more expensive image generation process. Furthermore, many images generated using diffusion models undergo post-processing to further edit the images, which was also out of the scope of our research.

When the synsets on ImageNet [15] did not contain a sufficient number of images, images from a different synset under the same subclass were used to ensure that each subclass had 5,000 images. This could have introduced biases to the trained model, which could affect the results of the study. Furthermore, using images of a higher resolution (e.g. 96× 96 pixels) was beyond our computational capabilities at that time and thus, was not explored.

Malicious actors may also use tools such as StealthDiffusion to make it more difficult to detect these images. This research has not explored how to combat against additional augmentations such as the usage of StealthDiffusion, cropping, flipping or compression. A CNN-based detector may be susceptible to adversarial attacks due to the nature of filters placing emphasis on the pixel-level features of the image, rather than global semantics. Possible defenses include using TRIM, a training-free method for combatting adversarial attacks [46]. Ensemble learning techniques could also be used, like those seen in MEXFIC [47], which harness the strengths of various models to further improve defenses from adversarial attacks. Furthermore, the rise of vision transformers [48] offers us further potential to detect synthetic images at scale [49].

## Conclusion

This research introduces the largest publicly available dataset of images generated by Stable Diffusion XL. Despite the existence of public datasets of over 1,000,000 images generated by diffusion models [26], there was no comparable dataset generated using SDXL. At 100,000 images, our dataset is orders of magnitude larger than similar public SDXL datasets, for example, *Stable Diffusion Face Dataset* [29] (3,000 images) and *Synthbuster* [27] (1,000 images).

This is the first attempt to generate an at-scale dataset for research usage using Stable Diffusion XL [14] It is also the first attempt to detect images generated by Stable Diffusion XL, expanding on the detection conducted by Wang et al. on various other diffusion models using DIRE [21]. The project builds on the research carried out by Bird and Lotfi in CIFAKE [11] by improving their test accuracy, testing on new image classes and assessing the model’s generalisability. This dataset has achieved a lower FID score than the three datasets produced by Borji [5,32]. As a result, it has been released to the public for further research.

The results were overwhelmingly positive, with 97.24% of the 20,000 images in the test set being correctly classified by the novel convolutional neural network detailed in Fig 4. Furthermore, the model was also found to be generalisable, with an accuracy of 92.04% on an unseen dataset, better than the accuracy of 90.1% achieved by ResNet-18 [16] on the same set. After assessing the performance on each subclass, it was revealed that the model could classify each class to a high accuracy, particularly with the car subclass, but less so with the spider subclass.

These results prove that it is still possible to detect images generated using diffusion models. Extending this research to create a detector suitable for general use will be the next step to recognising misinformation on a large scale.
